# Excitability, Inhibition, and Neurotransmitter Levels in the Motor Cortex of Symptomatic and Asymptomatic Individuals Following Mild Traumatic Brain Injury

**DOI:** 10.3389/fneur.2020.00683

**Published:** 2020-07-14

**Authors:** Alia L. Yasen, Miranda M. Lim, Kristianna B. Weymann, Anita D. Christie

**Affiliations:** ^1^Department of Human Physiology, University of Oregon, Eugene, OR, United States; ^2^Sleep Disorders Clinic, VA Portland Health Care System, Portland, OR, United States; ^3^Departments of Neurology, Behavioral Neuroscience, Medicine, and Oregon Institute of Occupational Health Sciences, Oregon Health & Science University, Portland, OR, United States; ^4^School of Nursing, Oregon Health & Science University, Portland, OR, United States; ^5^Faculty of Health Sciences, School of Kinesiology, Western University, London, ON, Canada

**Keywords:** mTBI, chronic symptoms, corticospinal excitability, intracortical inhibition, neurotransmitters

## Abstract

**Purpose:** The purpose of this study was to determine the level of excitability and inhibition, as well as the concentrations of excitatory and inhibitory neurotransmitters, in the motor cortex of individuals with acute and chronic symptoms from mTBI.

**Methods:** Fifty-three individuals were assigned to one of four groups: (i) without history of mTBI (Control), (ii) within 72-h of diagnosis of mTBI (Acute), (iii) with history of mTBI and no remaining symptoms (Chronic Asymptomatic), and (iv) with chronic symptoms from mTBI, lasting at least 3 months post-injury (Chronic Symptomatic). Measures of corticospinal excitability and inhibition were obtained using transcranial magnetic stimulation (TMS). On the same day, measures of glutamate and GABA concentrations were obtained from the primary motor cortex (M1) using proton magnetic resonance spectroscopy.

**Results:** MEP amplitude and area were both significantly lower in the Chronic Symptomatic group compared to the Control and Chronic Asymptomatic groups (*p* ≤ 0.05). Intracortical inhibition was not significantly different among groups (*p* = 0.14). The concentration of glutamate in M1 was similar between groups (*p* = 0.93) while there was a trend for a lower concentration of GABA in the Chronic Symptomatic group compared to the Acute group (*p* = 0.06).

**Conclusions:** Individuals with chronic mTBI symptoms appear to have lower corticospinal excitability compared with acutely-injured individuals and asymptomatic controls, but the absence of differences in intracortical inhibition, and concentrations of excitatory and inhibitory neurotransmitters in M1 suggests that neurotransmitter changes in the human brain post-mTBI do not follow the pattern typically seen in the animal literature.

## Introduction

Mild traumatic brain injury (mTBI) has been described as a diffuse injury that can impact both cognitive and motor function (1–4). However, an individual's physiological response to mTBI is often unpredictable, as there is a high degree of inter-individual variability in symptoms experienced (5). While symptoms recover within ~2 weeks for most individuals (6), 10–15% of the population with mTBI will continue to experience symptoms chronically (7). While acutely-injured individuals have consistently demonstrated a different neurophysiological profile compared with healthy controls (3, 8, 9), similar assessments in individuals with chronic symptoms have not been made. Therefore, little is known about the physiological characteristics of chronic mTBI and how they compare with characteristics of acute injury.

Work in rodent models of mTBI suggests that immediately after a mechanical injury to the head, the brain enters a state of excitation as excitatory neurotransmitters, such as glutamate, are released (10). Glutamate transport decreases following mTBI, allowing excess glutamate to stay in the synapse and prolong the excitotoxic environment in the brain. Following the initial excitatory phase, an inhibitory, “spreading depression” phase occurs as the brain attempts to maintain homeostasis (10). Studies show that neurons injured following mTBI demonstrate a reduction in dendrite length and inhibited neuronal signaling, suggesting that mTBI may lead to a change in the excitatory/inhibitory balance post-injury (11).

Indirect evidence from studies using transcranial magnetic stimulation to activate the first dorsal interosseous (FDI) muscle suggests the potential for alterations in corticospinal excitability and inhibition acutely following injury in humans as well. While lower excitability in individuals with mTBI has been documented (12), others report no difference (3), or greater excitability (13) acutely following injury. While the hyper-excitable state in animal models tends to resolve within hours after injury (10), the timeline in humans is unknown. Therefore, these differences in excitability across studies are likely attributed to the different post-injury times at which the measurements are obtained. Cortical inhibition, however, has consistently been shown to be greater in acutely-injured individuals compared with healthy controls, a finding which persists beyond symptom resolution (3, 13, 14). However, to our knowledge, similar assessments in individuals with chronic symptoms from mTBI have not been obtained.

Direct measurement of brain neurotransmitters, as reported in rodent models of the neurometabolic cascade, are challenging to obtain in humans. However, the use of proton magnetic resonance spectroscopy (^1^H-MRS), a non-invasive, reliable (15) technique, has produced a limited number of reports on neurotransmitter concentrations in individuals with mTBI. Using this technique, it has been shown that individuals with mTBI have altered neurometabolic profiles in comparison to controls (9). Specifically, individuals with mTBI have been reported to have lower levels of glutamate, the primary excitatory neurotransmitter in the primary motor cortex (9) and dorsolateral prefrontal cortex (16). Measures of GABA, the primary inhibitory neurotransmitter, have been shown to be lower in individuals with mTBI, compared with controls, in the dorsolateral prefrontal cortex (9, 16), but not in the primary motor cortex (16–18). These previous studies, however, involved measurements taken from those with acute injury (9) and asymptomatic controls (18). Assessments from individuals with chronic symptoms from mTBI will substantially enhance our understanding of the neurophysiological recovery from mTBI.

The purpose of this study was to determine the level of excitability and inhibition, as well as the concentrations of excitatory and inhibitory neurotransmitters, in the motor cortex of individuals with acute and chronic symptoms from mTBI. It was hypothesized that (i) acutely-injured individuals would demonstrate higher levels of corticospinal excitability and higher levels of intracortical inhibition than healthy control participants, as assessed in the FDI; (ii) individuals with chronic symptoms from mTBI would experience similar levels of corticospinal excitability and higher levels of intracortical inhibition compared with healthy control participants, as assessed in the FDI; (iii) acutely-injured individuals would have higher glutamate and GABA concentrations in the motor cortex than healthy control individuals; and (iv) individuals with chronic symptoms from mTBI would have similar levels of glutamate in the motor cortex, but elevated levels of GABA compared to healthy control participants.

## Materials and Methods

### Participants

Fifty-three individuals were consented and enrolled in the study under IRB approval. Individuals were assigned to one of four groups: (i) without history of mTBI (Control, *n* = 11, six females), (ii) within 72-h of diagnosis of mTBI (Acute, *n* = 9, five females), (iii) with history of mTBI and no remaining symptoms (Chronic Asymptomatic, *n* = 20, 10 females), and (iv) with chronic symptoms from mTBI, lasting at least 3 months post-injury (Chronic Symptomatic, *n* = 13, 6 females). All injuries met the definition of concussion provided by the 4th International Consensus Statement on Concussion in Sport (19). All mTBIs, acute and chronic, had been diagnosed by health professionals (certified athletic trainer or physician). The existence or absence of chronic symptoms was self-reported. All participants provided written informed consent and were asked to complete a brief medical history and TMS and MRI safety screening questionnaires to determine eligibility to participate in each portion of the study.

Exclusion criteria for all participants included: [1] history of cognitive deficiencies, such as permanent memory loss or concentration abnormalities (unrelated to the mTBI injury), [2] loss of consciousness from the mTBI lasting more than 1 min, [3] history of attention deficit hyperactivity disorder, or (4) contraindications to the use of TMS (20) or MRS. All procedures were reviewed and approved by the University of Oregon Institutional Review Board prior to any data collection. Three individuals (two from the Chronic Symptomatic group and one from the Chronic Asymptomatic group) were excused from the TMS portion of the study due to contraindications, leaving a total of 50 participants for TMS analysis. Three different individuals (one each from the Control, Chronic Asymptomatic, and Chronic Symptomatic groups) were excused from the MRS portion of the study due to contraindications, leaving a total of 50 participants for MRS analysis.

Participants in the Acute group were tested within 72 h of sustaining their mTBI (±2 days). Participants in the Chronic Symptomatic group were tested an average of 3.4 years post-injury (± 2.1 years), and those in the Chronic Asymptomatic group were tested an average of years 4.3 years post-injury (± 2.9 years).

### Measurement Schedule

Each participant completed one testing session, during which measures of corticospinal excitability and inhibition were obtained using TMS. On the same day, measures of glutamate and GABA concentrations were obtained using proton magnetic resonance spectroscopy (^1^H-MRS) from the primary motor cortex (M1). The order of testing (TMS and MRS) was pseudo-random and was determined by a combination of the participant's schedule and the availability of the MRI facility. In preliminary analysis, there was no systematic difference in measures based on the order of testing. Individuals in the acute mTBI group were tested within 72 h of sustaining their injury (± 2 days).

### Electromyography (EMG)

A preamplified bipolar, Ag-AgCl EMG electrode (DE-2.1, Delsys Inc., Boston, MA), with an inter-electrode distance of 1 cm was placed over the first dorsal interosseous (FDI) of the dominant hand. This electrode was connected to a portable amplifier (Delsys Inc., Boston, MA), which further amplified and band-pass (20–450 Hz) filtered the EMG signal. A ground electrode was secured to the posterior aspect of the distal ulna. The EMG signal was sampled at 5 kHz with a 16-bit A/D converter (NI USB-6251, National Instruments, Austin, TX).

### Motor Evoked Potential (MEP) and Cortical Silent Period (CSP)

TMS was performed using a flat, 70-mm figure-of-eight coil positioned over the optimal site of the contralateral motor cortex to elicit motor evoked potentials (MEP) in the dominant FDI. The optimal site was defined as the position that yielded the largest MEP consistently. Once the optimal site was located, the resting motor threshold (RMT) was determined as the lowest intensity required to evoke a response of at least 50 μV in at least five out of 10 trials (21, 22). The number of stimulations performed to determine RMT was not recorded, but generally ranges between ~20–30 stimuli. A rest period of at least 5 s was provided between each stimulation. Eight cortical silent periods were then evoked by single-pulse stimulations delivered at 120% of the RMT while participants maintained an isometric of the dominant FDI at 50% of their maximal voluntary contraction (MVC). MVC force of the FDI was quantified using a force transducer (MBP-5; Interface, Scottsdale, AZ) placed against the lateral aspect of the second digit on the dominant hand. Participants were instructed to push against the transducer as hard as possible two times, with 2 min of rest between trials. The highest value was used as the MVC measure. Visual feedback, including a target line at 50% MVC, was provided to the participants and the experimenter ensured that participants had reached the target level of EMG prior to providing the TMS pulse. A rest period of 30 s was provided between stimulations.

**Figure 1:**
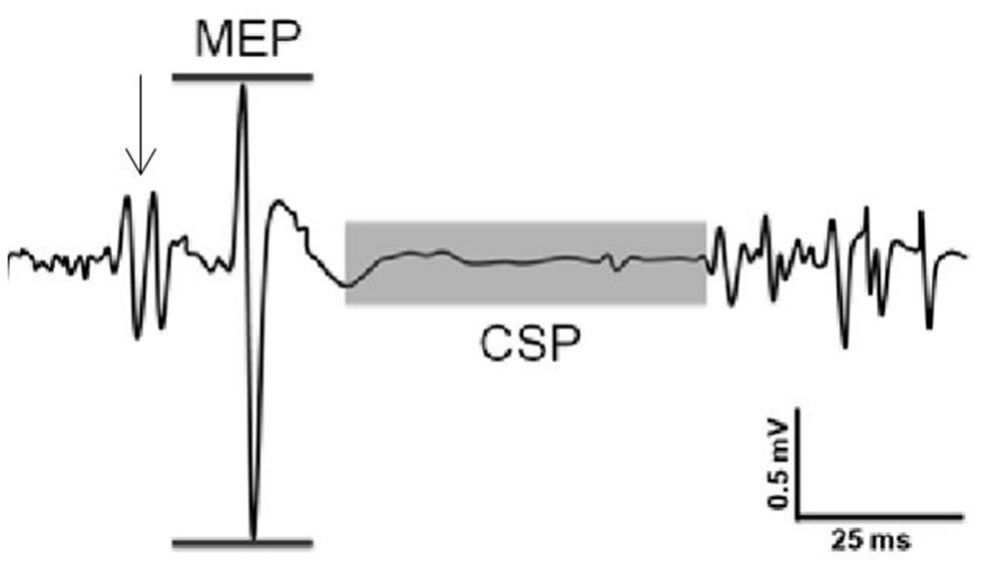
Sample recording of evoked responses. Excitability was assessed through the peak-to-peak amplitude of the MEP (horizontal lines). Inhibition was assessed through the cortical silent period (CSP) duration (shaded area). Arrow indicates stimulation from TMS.

Corticospinal excitability was assessed through the peak-to-peak amplitude of the active MEP (Figure 1). The area under the MEP, calculated from the rectified EMG signal, was also calculated as a secondary measure of corticospinal excitability. Inhibition was assessed through the CSP duration and was manually identified for each trial as the time between the end of the MEP and the resumption of voluntary EMG activity (Figure 1). All trials were analyzed, using a custom-written MATLAB (Mathworks Inc., Natick, MA) program, by the same trained investigator, who was blinded to the participants' group at the time of data analysis. Such manual selection of EMG onset times has been shown to have similar reliability as automated procedures (23).

### Magnetic Resonance Spectroscopy

^1^H-MRS was performed in a 3T whole-body MR scanner (Skyra: Siemens, Erlagen, Germany) using a 32-channel receive-only phased-array head coil. Anatomical MRI images of the brain were acquired and reviewed by the MRI technologist, and no incidental findings or brain abnormalities were reported for any of the participating subjects.

### Functional Localizer Task

**Figure 2:**
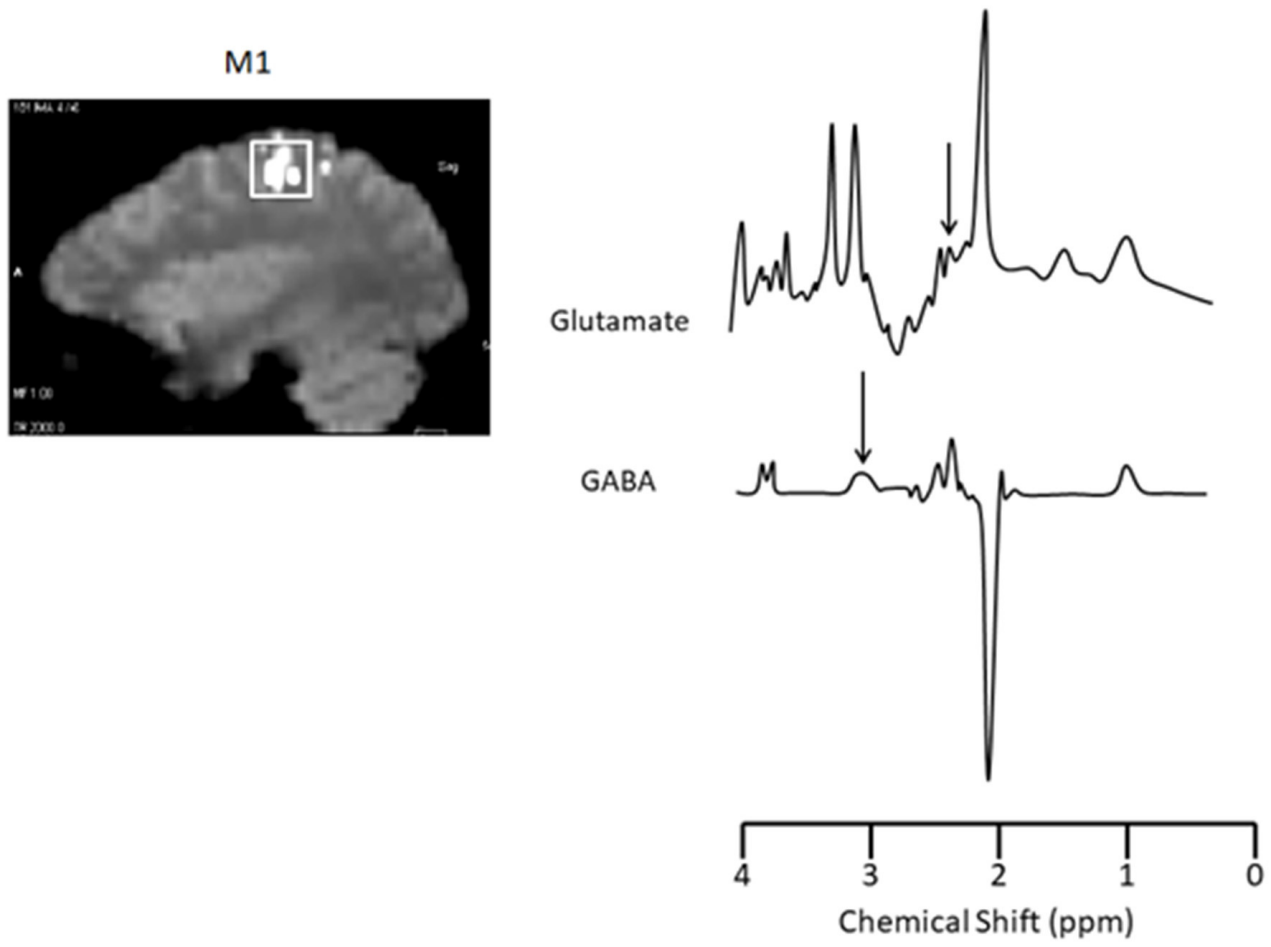
Functional localizer scans for voxel placement and Glutamate and GABA sample spectra from M1. Left: M1 localizer scan (voxel size: 20 × 20 × 20 mm). Right: The area under glutamate (2.4 ppm; TR/TE = 1,500/30 ms, 256 acquisitions) and GABA (3.0 ppm; TR/TE = 2,000/68 ms, 128 acquisitions) peaks were calculated and expressed relative to total creatine. Target peaks for glutamate and GABA are indicated by an arrow.

A functional localizer task was administered in order to place a voxel at M1. During the M1 localizer task, participants were asked to tap the index finger of their dominant hand on the MRI bed upon visual presentation of a target word. Participants alternated between tapping for 24 s and resting for 24 s for ~3 min. Once the localizer task was complete, a voxel measuring 20 × 20 × 20 mm was placed in the region activated by the localizer contrast representing M1 (Figure 2, left).

### Glutamate and GABA Estimation

A 6-min single-voxel PRESS sequence (TR/TE = 1,500/30 ms, 256 acquisitions) (9) was used to assess glutamate and a 9-min adapted MEGA-PRESS sequence for GABA (TR/TE = 2,000/68 ms, 128 acquisitions) (24). Averaging spectra across theses sequences produce a signal:noise ratio sufficient for identifying the respective peaks (9, 24). Sample spectra are shown in Figure 2, right. The area under glutamate (2.4 ppm) and GABA (3.0 ppm) peaks were calculated and expressed relative to total creatine using LCModel (25). Creatine values did not change over time in M1 (*p* = 0.25). Therefore, we are confident that any differences in metabolic ratios expressed over creatine between groups or over time are a result of changes in glutamate or GABA rather than creatine.

### Statistical Analysis

One-factor (mTBI group) ANOVAs were used to assess differences in: age, height, weight, MEP amplitude, CSP duration, and glutamate and GABA concentration across mTBI groups. Where necessary, *Post-hoc* pairwise comparisons with Bonferroni adjustments were made. ANOVA data are presented as mean ± SD. For individuals in the Acute, Chronic Symptomatic and Chronic Asymptomatic groups, the relationships between time since injury and each of glutamate, GABA, MEP amplitude and CSP duration were determined using linear regression analyses.

## Results

### Participant Characteristics

Participant characteristics are presented in Table 1. There were no group differences in height (*p* = 0.21) or weight (*p* = 0.28), but the Chronic Symptomatic group was significantly older than the other three groups (*p* = 0.009).

**Table 1:** Group characteristics.

	**Control**	**Acute**	**Chronic asymptomatic**	**Chronic symptomatic**	***p***
*n*	10 (6 f)	9 (5 f)	19 (10 f)	12 (6 f)	
Age (years)	21.1 ± 0.9	20.8 ± 2.3	20.6 ± 2.0	26.7 ± 9.5	0.009
Height (cm)	169.9 ± 10.7	174.4 ± 11.1	173.7 ± 8.3	164.6 ± 7.3	0.21
Mass (kg)	66.1 ± 9.8	73.2 ± 14.2	72.5 ± 14.3	62.0 ± 12.4	0.28

### Motor Evoked Potential (MEP)

**Figure 3:**
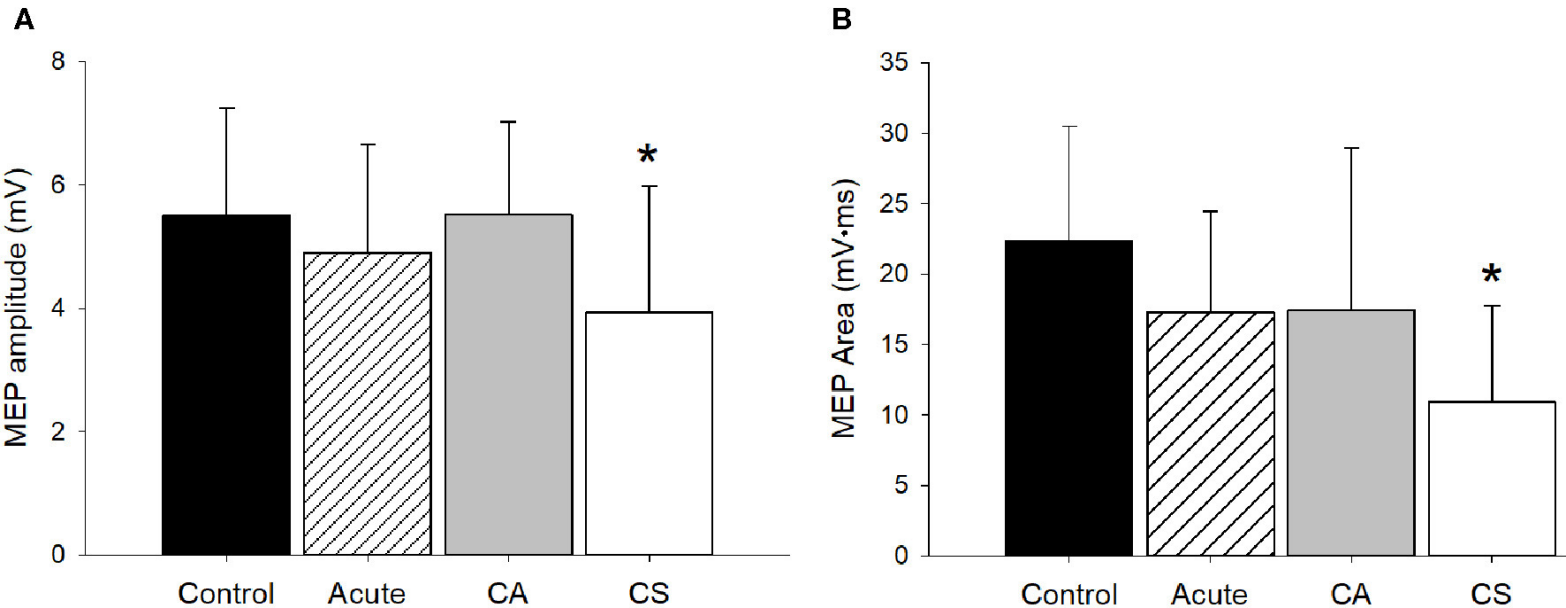
MEP amplitude and area. **(A)** The MEP amplitude was significantly different among groups (*p* = 0.05). *Post-hoc* comparisons revealed that the CS group had a significantly smaller MEP amplitude compared to the Control (*p* = 0.02) and CA (*p* = 0.02) groups. **(B)** The MEP area was significantly different among groups (*p* = 0.007). *Post-hoc* comparisons revealed that the CS group had a significantly smaller MEP amplitude compared to the Control (*p* = 0.006) and CA (*p* = 0.05) groups. CA, Chronic Asymptomatic; CS, Chronic Symptomatic. *Significantly different from Control and CA groups.

Both the MEP amplitude (*p* = 0.05) and area (*p* = 0.007) were significantly different between groups (Figure 3). *Post-hoc* comparisons revealed that the Chronic Symptomatic group had a significantly smaller MEP amplitude and area compared to the Control (*p* = 0.02, *p* = 0.06, respectively) and Chronic Asymptomatic (*p* = 0.02, *p* = 0.05, respectively) groups, indicating lower cortical excitability in the Chronic Symptomatic group. MEP amplitude was not significantly associated with time since injury (*r*^2^ = 0.01, *p* = 0.93). Data from three participants in the Chronic Asymptomatic group were found to be outliers and removed from the analysis (data from 20 total Chronic Asymptomatic participants were used in this analysis).

### Cortical Silent Period (CSP)

**Figure 4:**
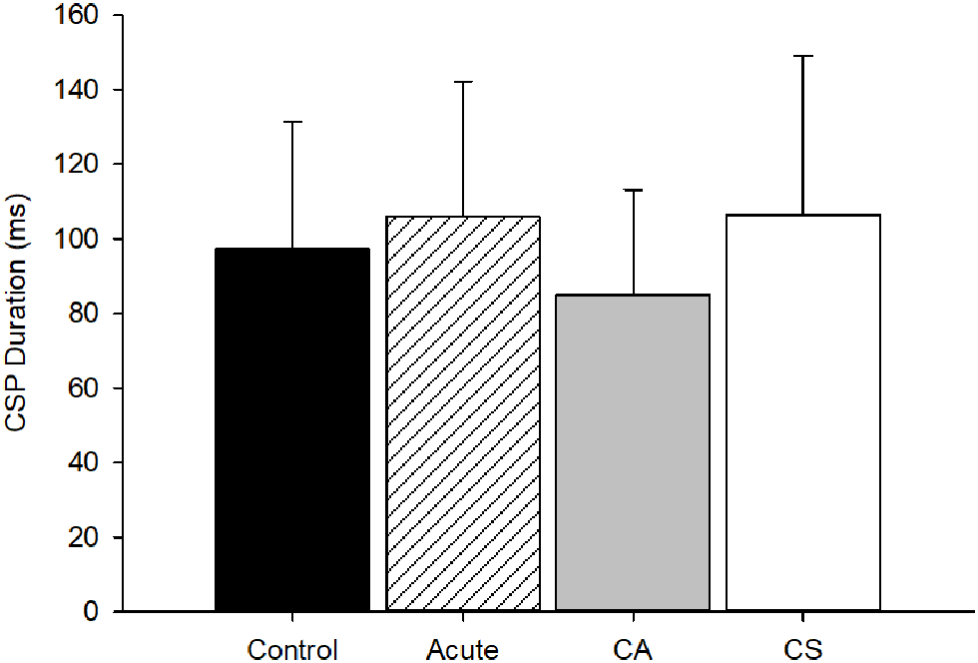
CSP duration group comparison. The CSP duration was not different among groups (*p* = 0.14). CA, Chronic Asymptomatic; CS, Chronic Symptomatic.

The CSP duration was not significantly different among groups (*p* = 0.14, Figure 4) indicating no effect of mTBI on intracortical inhibition. There was no significant relationship between CSP duration and time since injury (*r*^2^ = 0.04, *p* = 0.76).

### Glutamate

**Figure 5:**
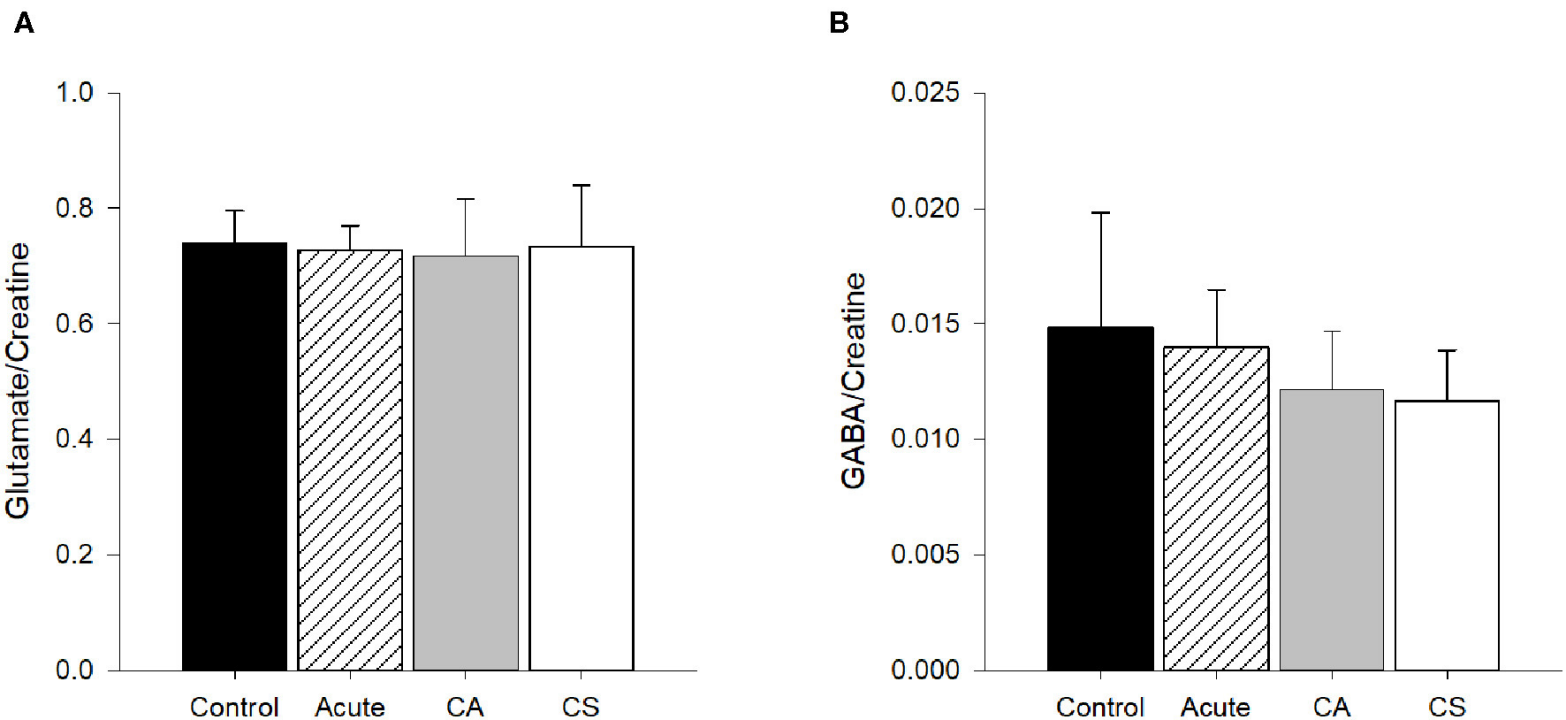
Glutamate and GABA concentrations/creatine in M1. **(A)** Glutamate/creatine concentrations were similar among groups (*p* = 0.93). **(B)** Concentrations of GABA/creatine were not quite statistically different among groups (*p* = 0.06). CA, Chronic Asymptomatic; CS, Chronic Symptomatic.

Glutamate/creatine values in M1 for all four groups are shown in Figure 5. Results from the ANOVA test indicated that glutamate/creatine values were similar among groups (*p* = 0.93). Glutamate concentrations were not significantly associated with time since injury (*r*^2^ < 0.01, *p* = 0.82).

### GABA

GABA/creatine values in M1 for all four groups are shown in Figure 4. Results from the ANOVA test indicated that GABA/creatine values were not quite statistically different between groups (*p* = 0.06). *Post-hoc* analysis indicated that the trend for significance was between the Acute and Chronic Symptomatic groups, where the Chronic Symptomatic group had lower levels of GABA in M1 compared to Acute participants (*p* = 0.04), with no other significant between groups differences (*p*≥0.20). There was no significant relationship between CSP duration and time since injury (*r*^2^ = 0.01, *p* = 0.91).

## Discussion

The purpose of this investigation was to determine the level of excitability and inhibition, as well as excitatory and inhibitory neurotransmitter concentrations, in the motor cortex of individuals with acute and chronic symptoms from mTBI, compared with controls. The results demonstrated that individuals with chronic symptoms from mTBI had lower MEP amplitudes than individuals in the two control groups, indicating lower corticospinal excitability. No differences in intracortical inhibition, as assessed by the cortical silent period, were observed between groups. There were no significant differences across groups in the concentration of glutamate or GABA, although there was a trend toward lower GABA in M1 of the Chronic Symptomatic group.

### Excitability

Consistent with some (3, 26), but not all (12) previous reports, corticospinal excitability, as assessed with MEP amplitude, was similar in acutely-injured individuals and controls. In the rodent model, the acute, excitatory phase of the Neurometabolic Cascade following mTBI tends to resolve fairly quickly (10). While the time course of this cascade is unknown in humans, it is possible that the initial release of excitatory neurotransmitters in the brain following mTBI may resolve in the human brain by 72-h post-injury. It is possible that differences in excitability in our Acute group were missed because too much time had elapsed between the initial injury and the data collection.

To our knowledge, this is the first study to report measures of corticospinal excitability in individuals with chronic symptoms from mTBI. In our data, chronically-symptomatic individuals showed smaller MEP amplitudes and areas, indicating lower corticospinal excitability, than those in the two control groups, but similar levels of cortical excitability to those with acute mTBI. This finding is consistent with reports in individuals with lasting symptoms following severe TBI, where a smaller MEP area compared with healthy controls has been documented (27). Our data suggest the lower corticospinal excitability is not simply an effect of the time since injury as there was no significant relationship between MEP amplitude and time since injury. It should be noted that, like other reports of corticospinal excitability following mTBI (e.g., 3, 26), the MEP amplitude was not normalized to an M-wave in this study. Although the non-normalized MEP value has been shown to be a reliable estimate of corticospinal excitability (28–30), an influence of differences in muscle properties across groups cannot be ruled out in our current results.

Based on animal literature regarding the relative timeline of metabolic changes in the brain post-mTBI, it was hypothesized that individuals with chronic symptoms from mTBI would be outside the window of time for excitotoxic changes to occur in the brain, and therefore, show no differences in cortical excitability compared to control groups. Following the initial stage of the Neurometabolic Cascade, however, the brain enters a “spreading depression” phase that may have longer-lasting effects than the acute excitotoxic phase (10). It is possible that individuals whose symptoms do not recover from mTBI after months or years may still be in this altered “depressed” stage of metabolic and/or ionic imbalance, resulting in the observed lower excitability of the corticospinal pathway.

It should be noted that the average participant in the Chronic Symptomatic group was significantly older (26.7 years) than participants in the other three groups (<22 years). Smaller MEP amplitudes in older individuals compared with young have been reported (31), however the average age of participants in this previous study was 63 years. Although participants in the Chronic Symptomatic group for this study were, on average, older than the other groups, the average age (~27 years) was still well below what is considered to be an “older adult” population. Therefore, it is unlikely that the age difference in this study substantially impacted the results.

### Inhibition

Based on previous studies documenting higher levels of intracortical inhibition both acutely and longer-term following mTBI (3, 26), it was hypothesized that both the Acute and Chronic Symptomatic groups would have higher levels of intracortical inhibition. However, we did not report any significant differences in CSP duration among groups. Although this comparison was not statistically different, the CSP duration was slightly higher in the Acute and Chronic Symptomatic groups compared to the two control groups, following a similar trend as previously reported in acutely-injured individuals (3, 13). This is a novel finding for individuals with chronic mTBI symptoms, warranting further investigation.

### Glutamate Concentrations

Evidence from animal studies suggests that the concentration of glutamate is higher in the brain immediately post-mTBI, but typically resolves within hours (10). In the current study, however, we found no difference in concentrations of glutamate in M1across groups. Although the MEP amplitude is thought to be mediated by glutamate (32), these results suggest that differences in glutamate concentrations do not explain our observation of lower corticospinal excitability in the Chronic Symptomatic group. One limitation of ^1^H-MRS is that it provides a total concentration of a neurotransmitter, but is not capable of distinguishing intra- and extra-cellular volumes. Therefore, it is possible that differences in the release or action of glutamate may result in lower MEP amplitudes, but would not detected using the ^1^H-MRS technique. Further work is therefore necessary to understand the mechanisms for the lower excitability in those with chronic symptoms from mTBI.

A lack of difference in the Chronic Symptomatic group is consistent with findings from (18), who found no difference in M1 concentrations of glutamate in athletes 3 years after an mTBI, although their participants were asymptomatic. The lack of a difference in the Acute group is in contrast with evidence suggesting lower levels of glutamate in M1, in athletes with mTBI, 1 week post-injury (9). We found no significant association between glutamate concentrations and the time post-injury, which is somewhat contradictory to a clearer time course of changes outlined in rodent models (10). However, in the rodent model the hyper-excitable state that is primarily mediated by glutamate resolves within hours post-injury. It is therefore possible that participants in our study were outside this time window. Further work is necessary to fully understand the time course in humans.

### GABA Concentrations

Few ^1^H-MRS studies have focused on GABA concentrations post-mTBI. We have previously shown no significant differences in GABA concentrations in M1 between acutely-injured individuals and healthy controls (16). Further, Tremblay et al. (18) found no differences in M1 GABA concentrations in their asymptomatic mTBI group 3 years post-injury. Similar to these previous studies, we found that GABA concentrations in M1 of the Acute and Chronic Asymptomatic groups was similar to that of uninjured controls.

To our knowledge, this is the first report of GABA concentrations in individuals with chronic symptoms post-mTBI. Although not statistically significant, we did observe a trend toward lower GABA concentrations in the Chronic Symptomatic group, relative to the Acute group. While this finding requires further investigation, it suggests the possibility that the neurochemical changes in the chronic post-injury phase may be different from those experienced acutely. Similar to our measures of glutamate concentrations, the intra- and extracellular concentrations of GABA cannot be determined with ^1^H-MRS. It is therefore possible that differences in the action of GABA may exist between groups, but would not have been detected in this study.

## Conclusions

We have shown that individuals with chronic mTBI symptoms appear to have lower corticospinal excitability compared with acutely-injured individuals and asymptomatic controls. However, no differences in intracortical inhibition were found across groups. Further, the lack of difference in glutamate and GABA across groups suggests that neurotransmitter changes in the brain post-mTBI did not follow the pattern typically seen in the animal literature. In humans, changes in glutamate and GABA concentrations in the brain may follow a different time-course, which should be investigated further.

## Data Availability Statement

The raw data supporting the conclusions of this article will be made available by the authors, without undue reservation.

## Ethics Statement

The studies involving human participants were reviewed and approved by University of Oregon Institutional Review Board VA Portland Health Care System Institutional Review Board. The patients/participants provided their written informed consent to participate in this study.

## Author Contributions

AY, ML, KW, and AC conceived and designed research, edited and revised manuscript, and approved final version of manuscript. AY and KW performed experiments. AY analyzed data. AY, ML, and AC interpreted results of experiments. AY prepared figures and drafted manuscript. All authors contributed to the article and approved the submitted version.

## Conflict of Interest

The authors declare that the research was conducted in the absence of any commercial or financial relationships that could be construed as a potential conflict of interest.
